# Simplified selective decontamination of the digestive tract reduces Gram-negative bloodstream infection and respiratory tract colonization in intensive care

**DOI:** 10.1186/cc11724

**Published:** 2012-11-14

**Authors:** Y Bar-Lavie, K Hussein, S Hadad, M Barzelay

**Affiliations:** 1 Rambam Medical Center, Haifa, Israel

## Background

Selective decontamination of the digestive tract (SDD) was shown to reduce acquisition of resistant bacteria and mortality in ICUs. A significant reduction of bacteremia, candidemia, ventilator-associated pneumonia, respiratory tract and rectal colonization have been found in multicenter, randomized studies. SDD was proven to be clinically safe and cost-effective in multiple clinical studies but has not been accepted by the critical care community as a standard of care. Reluctance to use SDD despite proof to the contrary is mostly explained by its perceived potential to raise bacterial resistance to parenteral and enteral antibiotics or rebound infection after their cessation. We studied the effect of a simplified SDD protocol on Gram-negative colonization of the respiratory tract and bloodstream infection.

## Methods

During 2011, all adult ICU patients on mechanical ventilation for more than 3 days were included. Enteral medication of neomycin and polymixin-E was given four times daily until gastric tube removal or ICU discharge. Blood, sputum cultures and rectal screening were taken on admission and biweekly until 3 days after ICU discharge. The emergence of multidrug-resistant (MDR) bacteria was investigated and compared with 2010. The in-hospital length of stay and 28-day mortality were compared.

## Results

Out of 506 patients, 277 (74% of eligible) received SDD during 2011, compared with none of 458 patients in 2010. There was a 40% reduction of positive blood cultures (*P *= 0.01) and a 27.3% reduction of positive sputum cultures with MDR bacteria (*P *= 0.04). There was a shift from MDR towards sensitive bacteria. This was mostly prominent in the *Acinetobacter*, *Klebsiella *and *Pseudomonas *species. There was no concomitant elevation of other pathogenes as MRSA, VRE, Clostridium or Candida. There was an 18.4% relative reduction in 28-day mortality and a 2-day reduction in median hospital length of stay (*P *= 0.006).

## Conclusion

The simplified SDD protocol reduced ICU acquisition of Gram-negative resistant infections without a rise in Gram-positives or fungi. The bacterial epidemiology of the unit changed shifting to sensitive bacteria, and the 28-day mortality and hospital length of stay have improved. This study supports the safety and efficacy of this SDD protocol and justifies its further investigation in more ICUs.

